# Inflammatory responses to metal oxide ceramic nanopowders

**DOI:** 10.1038/s41598-021-89329-7

**Published:** 2021-05-18

**Authors:** Shannon Jamieson, Amy Mawdesley, David Deehan, John Kirby, James Holland, Alison Tyson-Capper

**Affiliations:** 1 Faculty of Medical Sciences, Translational and Clinical Research Institute, Newcastle University, Newcastle upon Tyne, NE1 4HH UK; 2 Musculoskeletal Unit, Department of Orthopaedics, Freeman Hospital, Newcastle upon Tyne, NE7 7DN UK

**Keywords:** Cell biology, Immunology

## Abstract

Ceramic orthopaedic implants are increasingly popular due to the need for robust total joint replacement implants that have a high success rate long-term and do not induce biological responses in patients. This study was designed to investigate the biological effects of ceramic nanopowders containing aluminium oxide or zirconium oxide to activate the human macrophage THP-1 cell line. In vitro investigation of pro-inflammatory gene expression and chemokine secretion was performed studied using RT-qPCR and ELISA, respectively. TLR4 inhibition, using a small-molecule inhibitor, was used to determine whether ceramic-mediated inflammation occurs in a similar manner to that of metals such as cobalt. THP-1 macrophages were primed with ceramics or LPS and then treated with ATP or ceramics, respectively, to determine whether these nanopowders are involved in the priming or activation of the NLRP3 inflammasome through IL-1β secretion. Cells treated with ceramics significantly increased pro-inflammatory gene expression and protein secretion which was attenuated through TLR4 blockade. Addition of ATP to cells following ceramic treatment significantly increased IL-1β secretion. Therefore, we identify the ability of ceramic metal oxides to cause a pro-inflammatory phenotype in THP-1 macrophages and propose the mechanism by which this occurs is primarily via the TLR4 pathway which contributes to inflammasome signalling.

## Introduction

Total joint replacement (TJR) is the gold standard treatment option for patients with end-stage osteoarthritis (OA) and aims to alleviate symptoms of chronic OA such as pain and joint stiffness which can worsen over time and lead to disability^1^. Total hip replacement (THR) is one of the most common types of TJR performed in the UK with 1,095,754 primary surgeries taking place between 2003 and 2018^2^, the majority of which were indicated for OA.

Traditionally, THR implants comprised of a metal femoral head and polyethylene acetabular cup which have high failure rates due to excessive material wear of polyethylene and subsequent osteolytic effects from polyethylene debris. Together this excessive wear and osteolysis means patients often experience aseptic loosening of the implant, pain and reduced mobility^3,4^ therefore requiring complicated revision surgery. The use of metals for THR implants remains common place with both the femoral head and acetabular cup most commonly made from a cobalt-chromium (CoCr) alloy in metal-on-metal (MoM) hip implants. These MoM implants can produce up to five-times less wear debris than their polyethylene counterparts^5^. However, metal debris has been linked to the development of inflammatory pseudotumours^6,7^. Pseudotumours are benign soft tissue masses containing a mixture of inflammatory cells e.g. macrophages and T cells, which localise to the site of the TJR implant. It has been estimated that pseudotumours can occur in up to 61% of patients that receive (MoM) implants^6,7^. The aetiology behind this response remains unknown, however, extensive research has shown that cobalt ions from CoCr implants are capable of inducing inflammatory changes in a monocyte cell line in vitro through a Toll-like receptor 4 (TLR4)-mediated mechanism^8^.

Patients requiring primary joint replacements are increasingly younger than in previous years due to higher average body mass indexes (BMI) putting extra pressure on joints as well as patients who lead very active lifestyles experiencing extensive wear of their joints. Additionally, with an ageing population and increased numbers of TJR surgeries taking place each year, there is a huge health economic burden of revision surgery as patients undergoing revision often require extended hospital stays and an increased risk of thromboembolic disease and infection^9^. Consequently, there is a growing need for low-wearing and therefore longer lasting implant material hence the use of ceramic THR implants is now increasing in popularity^2^. Ceramics are ‘tough’ materials which are extremely low-wearing and thought to be bio-inert which in theory would elicit less wear-associated inflammatory reactions and reduce the need for revision surgery. The most commonly used ceramic implants in the UK are the Ceramtec BIOLOX *delta* which are comprised of Zirconium-toughened Alumina (ZTA) with the respective percentages of each ceramic being approximately 18% and 82%^10^.

A recent case study from a patient who had received a ceramic-on-ceramic (CoC) THR implant presented with pain and a loud squeaking noise in their hip five years post-surgery. Upon revision, analysis of the CoC implant revealed extensive wear equivalent of 10 mm^3^ per day for 1 year despite no issues with alignment or periprosthetic fracture^11^. Another case study published recently described the presence of an inflammatory pseudotumour in a 54-year old patient 6 years after receiving a CoC THR implant^12^. Together, these reports suggest that ceramics may not be as bio-inert as initially thought and extensive wear of these implants is still possible despite the notion that they are low-wearing. It is therefore important to investigate potential inflammatory responses to ceramics as their popularity continues to rise it becomes more likely that cases like those discussed occur more commonly.

Previous studies have shown that peripheral blood mononuclear cells (PBMCs) cultured on ceramic surfaces show increases in pro-inflammatory interleukin-6 (IL-6) and interleukin-1beta (IL-1β) secretion, a finding which was also mirrored by the NIH3T3 fibroblast cell line^13^. However, conflicting research has shown that other common inflammatory markers such as tumour necrosis factor-alpha (TNF-α) were not increased in the THP-1 monocytic human cell line following treatment with aluminium oxide^14^. These studies demonstrate clear gaps in the knowledge of inflammatory responses to ceramics and provide evidence that more research is required to improve our understanding of the immunobiology of these materials.

TLR4 has been implicated in inflammatory and endothelial responses to cobalt^8,15^ and the use of a TLR4 neutralising antibody has been shown to decrease interleukin-8 (IL-8), a pro-inflammatory and neutrophil attractant protein, from a monocyte cell line^16^. However, additional research has shown that use of a TLR4 antagonist does not cause a decrease in IL-1β secretion^17^. Mature IL-1β secretion requires activation of the nod-like receptor pyrin-containing domain 3 (NLRP3) inflammasome pathway. The pathways involved in inflammasome activation are complex and in the case of the NLRP3 inflammasome responses can be induced by a number of different stimuli and for complete activation two signals are required. The first signal, known as ‘priming’, is induced by extracellular stimuli such as pathogen-associated molecular patterns (PAMPs) which activate pattern recognition receptors (PRRs) such as TLRs resulting in NF-κB activation. Activation of NF-κB up-regulates the expression of both NLRP3 and pro-IL-1β required for NLRP3 receptor activation^18^. The second signal required for full inflammasome activation is usually induced by detection of a damage-associated molecular pattern (DAMP) such as extracellular ATP released from damaged or lysed cells. This causes the cleavage of pro-caspase-1 to its active form. The active caspase-1 enzyme can then cleave pro-IL-1β causing the mature form to be secreted from the cells. Mature IL-1β can then mediate pro-inflammatory effects on nearby cells by inducing the transcription of genes which result in acute inflammation. Together, this pathway is termed the ‘canonical’ inflammasome and is complimented by a ‘non-canonical’ inflammasome which acts in a similar way and utilises the enzymes capase-4 and caspase-5 to induce pyroptosis in humans^19^. Research by Caicedo et al., demonstrated the ability of cobalt, chromium, and nickel ions as well as CoCr particles to upregulate IL-1β secretion in a caspase-1-dependent manner in human macrophages^20^. Moreover, Samelko et al., published findings in agreement to this and demonstrated the importance of the NLRP3 inflammasome pathway in CoCr particle-mediated inflammatory responses when compared to TLR4^17^. It is therefore important to consider both the role of TLR4 as well as that of the NLRP3 inflammasome when considering biologic responses to wear debris including both metals and ceramics.

Another effect of inflammasome activation is rapid and pro-inflammatory cell death known as pyroptosis. A distinct form of cell death, pyroptosis is characterised by caspase-1 mediated cell lysis through cleavage of gasdermin D (GSDMD) which forms pores on the plasma membrane allowing the release of cellular contents^21^. NLRP3 activation has been shown to increase caspase-1 activity and therefore is of interest when considering host responses to wear debris as caspase-1 has been shown to have an important role in mediating pro-inflammatory responses to materials used in joint replacement such as polymethylmethacrylate (PMMA) in human monocytes^22^. Due to the inflammatory nature of pyroptosis there is a need to investigate the effects of wear debris particles, including ceramics, on macrophages in order to better understand the pro-inflammatory mechanisms resulting in the pathology observed in patients.

There have been clear links between inflammation and OA demonstrated in a number of studies. Chemokine C–C ligand 2 (CCL2) is a chemoattractant cytokine which can be secreted by a number of cell types including macrophages and acts as a mediator of monocyte recruitment. Investigations of the role of CCL2 in knee injury have demonstrated that patients with higher synovial fluid CCL2 concentrations also exhibit higher clinical pain scores^23^ as well as the potential link between CCL2 gene mutations and the development of OA^24^. Similarly to CCL2, another monocyte-specific chemoattractant cytokine is chemokine c–c ligand 3 (CCL3) and investigations of CCL3 have shown the potential for plasma CCL3 levels to be used a potential biomarker in order to detect and diagnose pre-radiographic OA in a Chinese cohort^25^. Chemokine c–c ligand 4 (CCL4) is another chemokine which has the capability to enhance monocyte migration and studies have shown that it is elevated in the synovial fluid of patients with OA when compared to a control group with articular ankle fracture^26^. CCL2, CCL3, and CCL4 secretion by human chondrocytes have also all been demonstrated in response to IL-1β^27^ therefore showing how there is a clear link between the ongoing pro-inflammatory mechanisms typical of OA. It is therefore extremely important to understand the effect that biomaterials have on the regulation of these pro-inflammatory proteins as further increases may manifest as adverse reactions and lead to aseptic loosening of an implant. A recent case study demonstrated elevated levels of macrophages and leukocytes present in soft tissue taken at the point of revision of a MoM THR implant. The patient had blood cobalt ion levels deemed within the safe range as well as increases in secretion of CCL3 and CCL4 from a human macrophage model in response to cobalt ions which can be attenuated through TLR4 inhibition^28^. However, interluekin-8 (IL-8), is a chemoattractant cytokine which is secreted by a number of cell types including macrophages and causes an increased migration of neutrophils during innate immune responses. Lawrence et al., demonstrated the ability of cobalt ions to increase IL-8 secretion in a human monocytic cell line which could then be abrogated through the use of a TLR4 small-molecule inhibitor^29^. Paish et al., recently reported chronic inflammation characterised by highly significant increases in IL-8, CCL2, CCL3 and CCL4 in the synovial fluid of a cohort of patients undergoing revision surgery following failed primary total knee replacement (TKR)^30^ therefore confirming the role of these pro-inflammatory chemokines in patient response. Together these findings present the clear links between inflammation in OA and inflammation in response to biomaterials, however, specific investigations of ceramic-mediated inflammatory responses remain limited.

By addressing the paucity in the literature as to the mechanism by which ceramics may induce an inflammatory and osteolytic immune response, novel therapies can be developed to prevent aseptic loosening and osteolysis in an increasing cohort of patients undergoing CoC THR.

## Results

### THP-1 cells are capable of phagocytosing ceramic nanopowders

**Figure 1:**
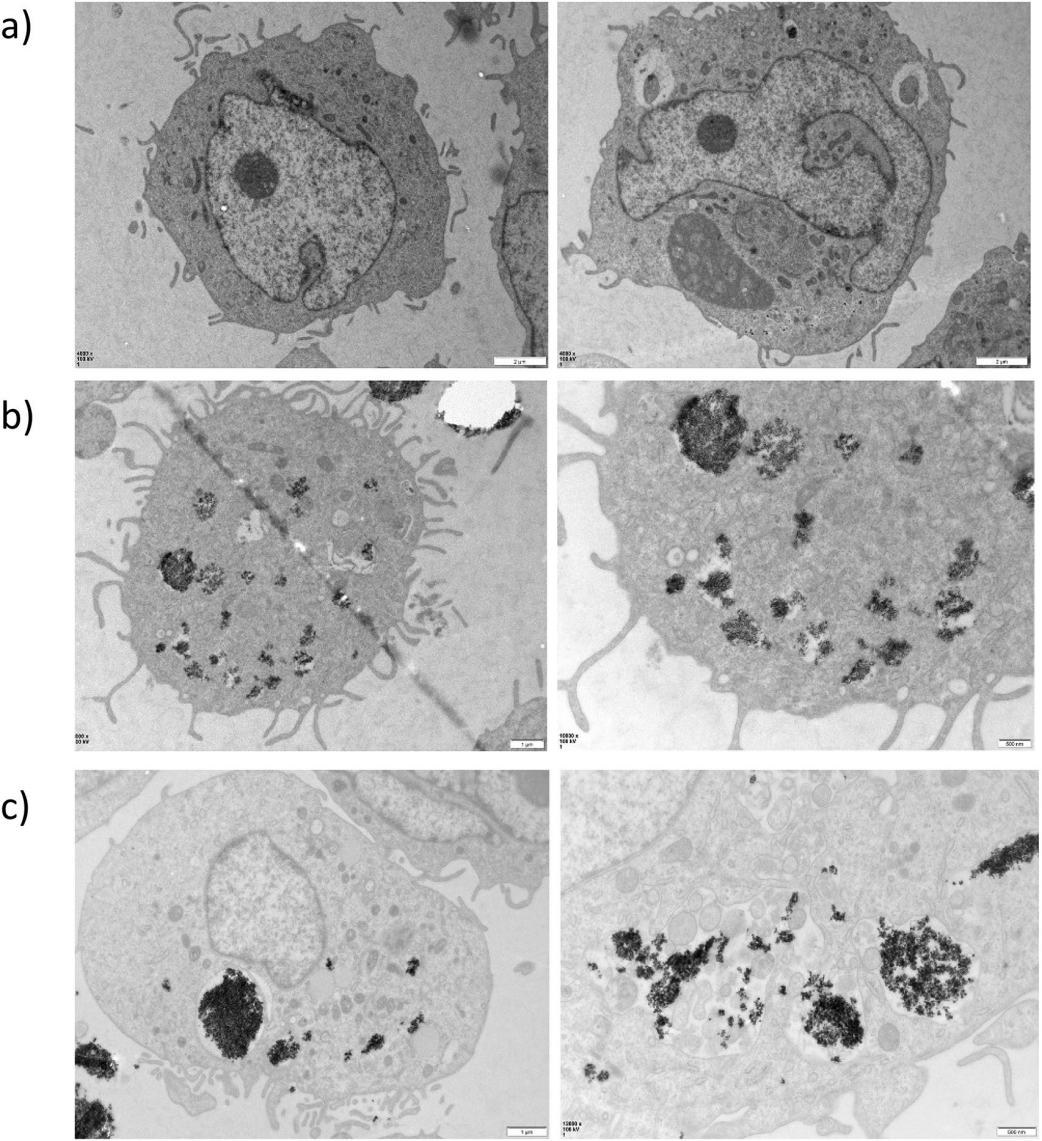
TEM images of THP-1 macrophages following metal oxide ceramic nanopowder treatment. THP-1 cells were activated to M1 macrophage phenotype using PMA and then either left untreated and given only fresh complete RMPI-1640 media or treated with ceramics for 24 h before being prepared and imaged using transmission electron microscopy. (**a**) Untreated THP-1 cells did not receive any dose of ceramic oxide nanopowders. These macrophages cells can be seen to have a characteristic large nucleus and formed pseudopodia, both of which are representative of an activated macrophage cell. (**b**) THP-1 cells treated with Al_2_O_3_ (50 μm^3^ particles per cell) for 24 h prior to TEM imaging. Dark areas of clumped staining show ceramic phagocystosed by the macrophage and held within distinct vacuole-like structures. (**c**) THP-1 macrophages treated with ZrO_2_ (50 μm^3^ particles per cell). Dense areas of dark staining show large distinctive clumps of ceramic which has been phagocytosed by the cell. Round vacuole-like structures surround the ceramic maintaining it within the cytoplasm.

THP-1 cells were treated with Al_2_O_3_ or ZrO_2_ for 24 h prior to fixation and TEM preparation. Cells were stained with heavy metals and TEM used to visualise cellular structures in order to determine whether THP-1 cells were capable of phagocytosing metal ceramic oxides. The TEM images presented here (Fig. 1) show clear evidence that the THP-1 cells can phagocytose ceramic nanopowders and can contain them within cytoplasmic vacuoles. Both the Al_2_O_3_ and ZrO_2_ nanopowders have been taken up by the THP-1 macrophages, although not all cells had phagocytosed the ceramics. This provides evidence for the capacity of macrophages to phagocytose metal oxide ceramics, therefore, subsequent investigations focussed on the effect that these biomaterials had on cell phenotype.

### Ceramic oxides induce no significant changes in cell viability and proliferation

**Figure 2:**
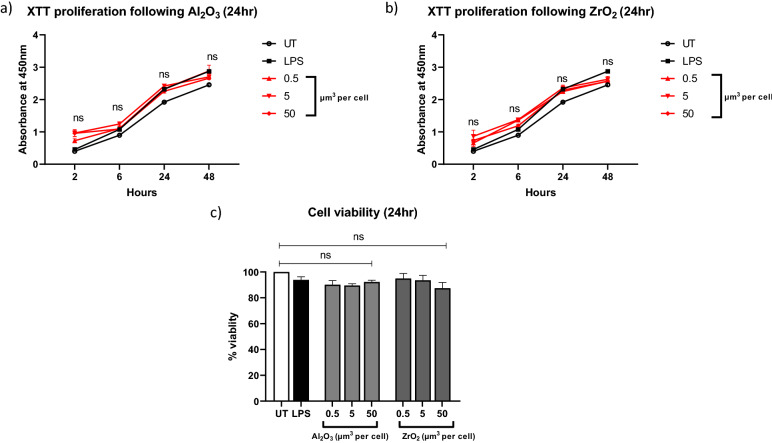
Cell viability and cellular proliferation following treatment with ceramic oxide nanopowders. THP-1 cells were treated for 24 h with 0.5–50 μm^3^ particles per cell Al_2_O_3_ or ZrO_2_ or LPS (10 ng/mL) before being assessed for viability and proliferation. (**a**) After treatment with Al_2_O_3_ for 24 h, cells were used in conjunction with the XTT platform and absorbance was measured on a nanoplate reader at 450 nm at 2–48 h timepoints. At each time point there was minimal difference between treatment groups and all groups following an increasing trend over the 48-h time course. The cells which received the highest dose of Al_2_O_3_ at 2 h (P = 0.0559), 6 h (0.2061), 24 h (P = 0.9996) and 48 h (P = 0.6275) saw no significant changes in absorbance following treatment with XTT. (**b**) Cells which were treated with ZrO_2_ for 24 h were then used for XTT proliferation analysis over a 2–48 h time course. ZrO_2_-treated cells saw no significant difference in absorbance when compared to untreated at 2 h (P = 02,867), 6 h (0.0577), 24 h (P = 0.9998), and 48 h (0.9714) at the highest dose. (**c**) Following a 24-h treatment with ceramic oxides or LPS, THP-1 cells were harvested and stained with trypan blue before being counted and normalised to the untreated control. There were no significant changes in cell viability between any treatment group and the control cells even at the highest concentrations of both Al_2_O_3_ (P = 0.5984) and ZrO_2_ (P = 0.1142). Graphs are represented of n = 3.

THP-1 cells were treated for 24 h with Al_2_O_3_ or ZrO_2_ (0.5–50 μm^3^ particles per cell) before cell viability was assessed using trypan blue or cellular proliferation was assessed using XTT. As seen in Fig. 2 there was no significant difference in viability of THP-1 cells treated with ceramics compared to untreated controls. The percentage change in cell viability when normalised to untreated was higher than 90% in all cases. XTT uses an electron coupling solution which can be reduced during cellular respiration causing a colour change. The colorimetric assay can then be read on a nanoplate reader at 450 nm using a time course to determine whether prolonged ceramic treatment effects cellular proliferation. There was no significant difference between the untreated THP-1 cells and those which received ceramic oxide nanopowders at 24 h followed by a 48-h XTT time course. Due to the lack of cytotoxic effects at 24 h this was therefore used as the treatment time in subsequent experiments.

### Ceramic metal oxides induce changes to inflammatory gene expression

**Figure 3:**
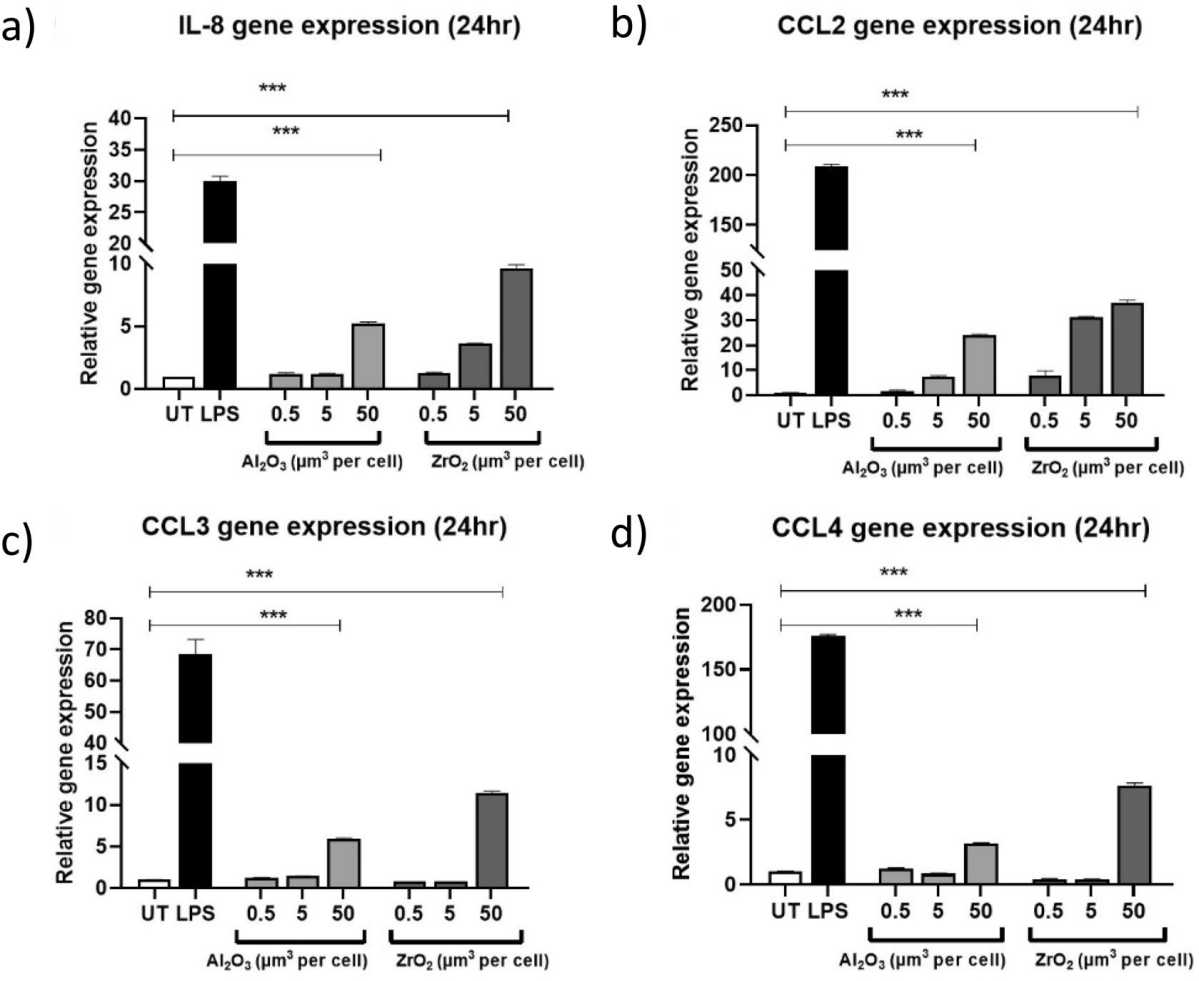
THP-1 macrophage relative gene expression following treatment with Al_2_O_3_ or ZrO_2_. THP-1 macrophages activated using PMA and treated with Al_2_O_3_ or ZrO_2_ (0.5–50 µm^3^ particles per cell) for 24 h. Relative gene expression was assessed using RT-qPCR. All experiments included an untreated negative control and LPS-treated (10 ng/mL) THP-1 macrophages as a positive control. (**a**) At the highest dosages of both ceramic metal oxide nanopowders there was a significant increase in the relative gene expression of *IL-8* when compared to untreated controls (Al_2_O_3_ P < 0.0001 and ZrO_2_ P < 0.0001). (**b**) *CCL2* relative gene expression increased significantly following treatment with 50 µm^3^ particles per cell Al_2_O_3_ (P < 0.0001) and ZrO_2_ (P < 0.0001) when compared to an untreated negative control. (**c**) There was significant increases in *CCL3* relative gene expression following treatment of THP-1 macrophages with 50 µm^3^ per cell Al_2_O_3_ (P < 0.0001) and ZrO_2_ (P < 0.0001). (**d**) THP-1 macrophages treated with 50 µm^3^ per cell Al_2_O_3_ or ZrO_2_ experienced significant increases in *CCL4* relative gene expression compared to an untreated control (Al_2_O_3_ P < 0.0001 and ZrO_2_ P < 0.0001). All data presented is representative of n = 3.

Previous investigations of metals have shown that monocyte cell lines (MonoMac-6) increase IL-8 gene expression following exposure to cobalt ions^8^. In this study, THP-1 cells were treated with Al_2_O_3_ or ZrO_2_ (0.5–50 µm^3^ particles per cell) for 24 h before RNA was extracted and cDNA synthesised. RT-qPCR was used and changes to relative gene expression are presented in Fig. 3. At the highest dosage (50 µm^3^ per cell) there was significant increase in the relative gene expression of *IL-8* (P < 0.0001), *CCL2* (P < 0.0001), *CCL3* (P < 0.0001) and *CCL4* (P < 0.0001) in both the Al_2_O_3_ and ZrO_2_-treated cells.

### Ceramic metal oxides induce increased inflammatory protein secretion

**Figure 4:**
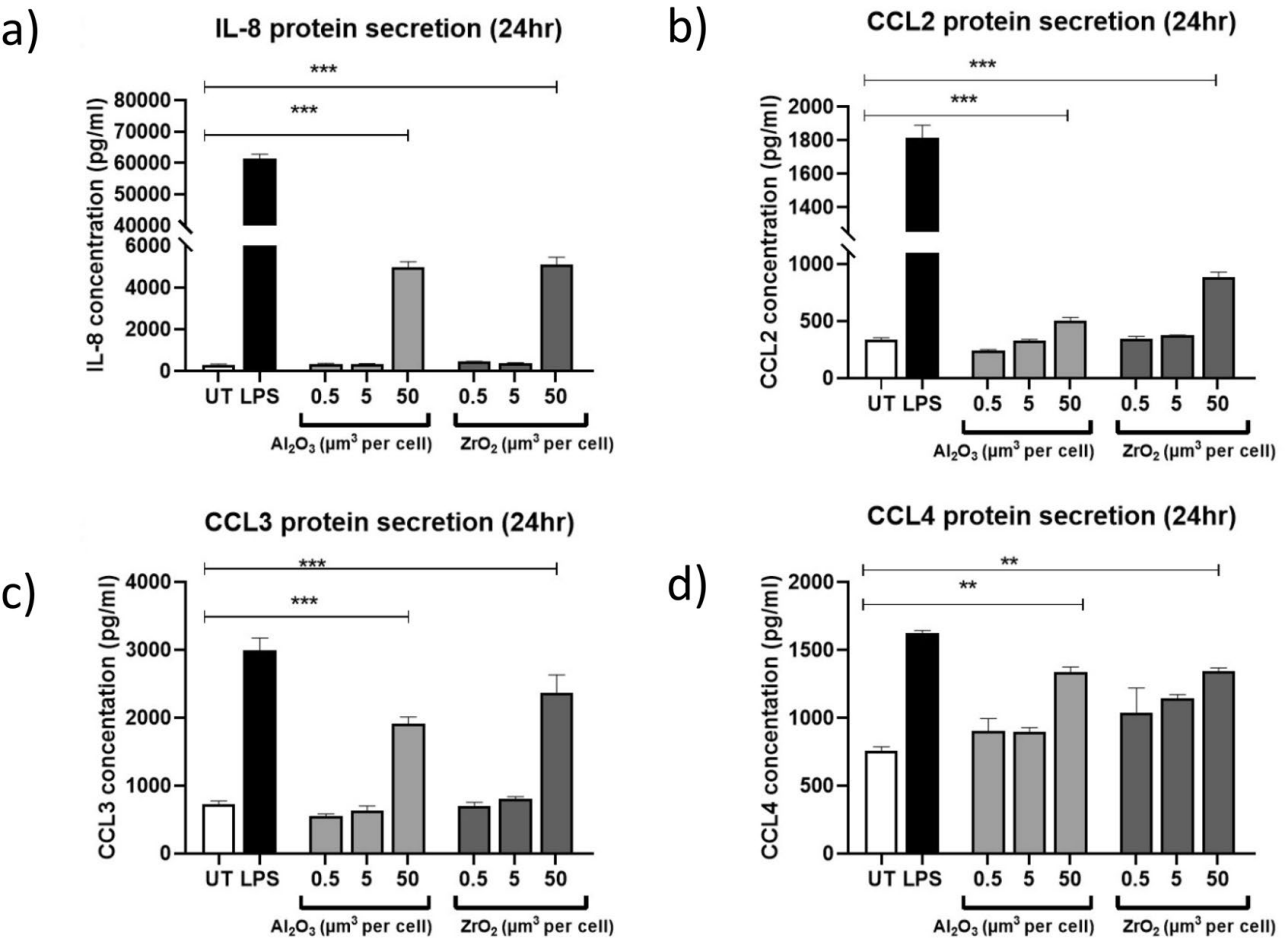
THP-1 macrophage protein secretion following treatment with metal ceramic oxide nanopowders for 24 h. THP-1 macrophages activated with PMA and treated with Al_2_O_3_ or ZrO_2_ (0.5–50 µm^3^ particles per cell) for 24 h. Supernatant was collected and used in conjunction with an ELISA platform to quantify protein secretion. In all cases, untreated cells were included as a negative control and LPS-treated (10 ng/mL) THP-1 macrophages were used as a positive control. (**a**) THP-1 macrophages experienced significant increases in the secretion of IL-8 following treatment with 50 µm^3^ particles per cell Al_2_O_3_ (P = 0.0002) and ZrO_2_ (P = 0.0001). (**b**) Both Al_2_O_3_-treated and ZrO_2_-treated THP-1 macrophages had significant increases in CCL2 protein secretion following a 24 h treatment time (both P < 0.0001). (**c**) CCL3 protein secretion increased significant after a 24 h incubation with 50 µm^3^ particles per cell Al_2_O_3_ (P < 0.0001) and ZrO_2_ (P < 0.0001). (**D**) At the highest dosage of Al_2_O_3_ and ZrO_2_ CCL4 protein secretion significantly increased compared to an untreated control (Al_2_O_3_ P = 0.0095) and ZrO_2_ (P = 0.0092). All data presented here is representative of *n* = 3.

Due to changes in relative gene expression observed, cells were treated for 24 h with Al_2_O_3_ or ZrO_2_ (0.5–50 µm^3^ particles per cell) and their supernatant collected. Supernatant was then analysed using ELISA (Fig. 4). At the highest dosages of both Al_2_O_3_ and ZrO_2_ there were significant increases in IL-8 (Al_2_O_3_ P = 0.0002 and ZrO_2_ P = 0.0001), CCL2 (P < 0.001), CCL3 (P < 0.001), and CCL4 (Al_2_O_3_ P < 0.0095 and ZrO_2_ P = 0.0092) secretion.

### TLR4-specific inhibition decreases inflammatory phenotype

**Figure 5:**
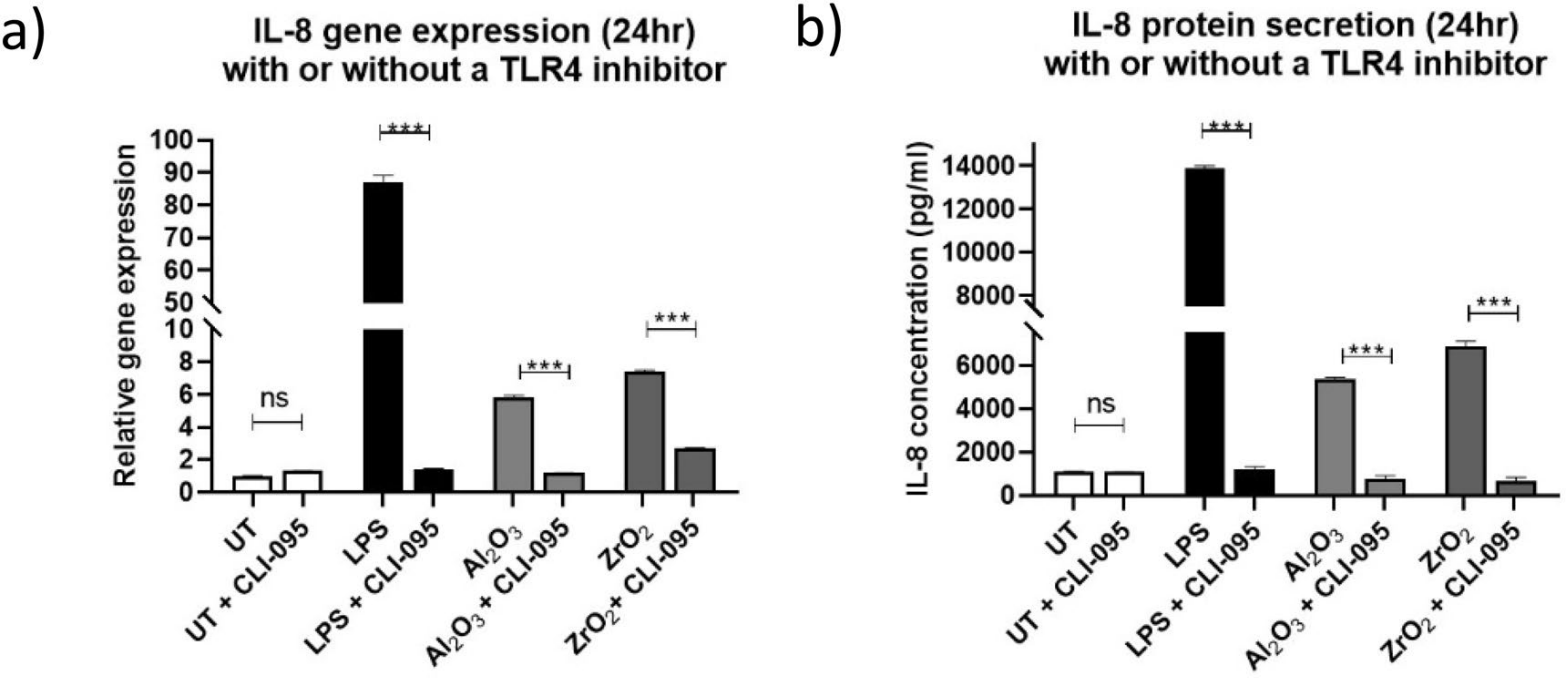
IL-8 relative gene expression and protein secretion following treatment with 50 µm^3^ per cell Al_2_O_3_ or ZrO_2_ with or without a pre-incubation with CLI-095. THP-1 macrophages were activated with PMA and pre-treated with CLI-095 for 6 h, a small-molecule inhibitor of TLR4, prior to a 24 h treatment with 50 µm^3^ particles per cell Al_2_O_3_ or ZrO_2_. (**a**) Relative gene expression of *IL-8* was assessed using RT-qPCR. THP-1 macrophages which were pre-treated with CLI-095 and then treated with 10 ng/mL LPS for 24 h experienced significant decreases in *IL-8* relative gene expression (P < 0.0001). Al_2_O_3_-treated cells which received CLI-095 also showed a significant decrease in *IL-8* relative gene expression compared to those which did not have a CLI-095 pre-treatment (P = 0.0070). There was a significant decrease in *IL-8* relative gene expression in cells which received CLI-095 followed by ZrO_2_ (P = 0.0065). (**b**) Supernatant was collected from THP-1 macrophages and used in conjunction with ELISA to quantify IL-8 protein secretion. There was a significant decrease in IL-8 protein secretion in cells which received CLI-095 followed by LPS (P < 0.0001). Al_2_O_3_ and ZrO_2_-treated cells which received a CLI-095 pre-treatment experienced significant decreases to IL-8 protein secretion compared to those which did not receive an inhibitor (Al_2_O_3_ P < 0.0001 and ZrO_2_ P < 0.0001). All data presented is representative of n = 3.

Inhibition of TLR4 has shown significant decreases in inflammatory gene expression in response to cobalt ions^16^. Therefore CLI-095, a small-molecule inhibitor of TLR4, was tested to determine whether changes to the inflammatory phenotype following ceramic treatment were TLR4 dependent. THP-1 cells were pre-treated with CLI-095 for 6 h before being treated with 50 µm^3^ particles per cell Al_2_O_3_ or ZrO_2_ for 24 h. The cells were lysed and RNA extracted for cDNA synthesis and RT-qPCR. Their supernatant was also collected for IL-8 ELISA analysis. As shown in Fig. 5, the cells which received 10 ng/mL LPS and CLI-095 experienced a significant decrease in *IL-8* gene expression (P < 0.0001) and protein secretion (P < 0.0001). Similarly, the Al_2_O_3_-treated cells saw significant decreases in *IL-8* gene expression (P < 0.0070) and protein secretion (P < 0.0001) in the CLI-095 pre-treatment groups. ZrO_2_-treated THP-1 cells which received CLI-095 also experienced significant decreases in IL-8 protein secretion (P < 0.0001) and gene expression (P < 0.0065).

### Metal ceramic oxides can induce secretion of IL-1β and inflammatory cell death

**Figure 6:**
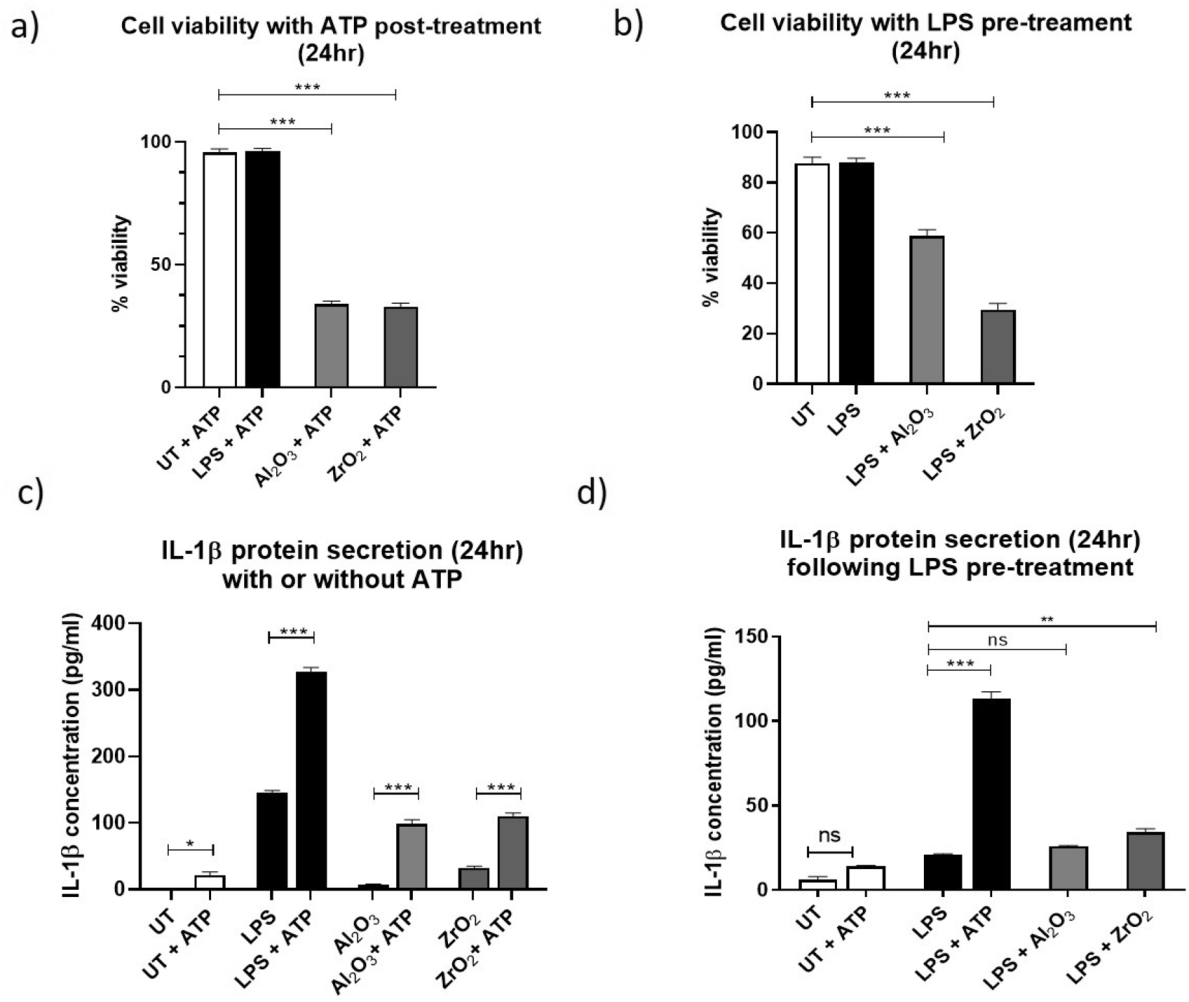
Cell viability following pyroptosis induction and IL-1β secretion and protein expression following induction of inflammasome activation with our without TLR4 blockade. (**a**) THP-1 macrophages activated using PMA and treated with 50 µm^3^ per cell Al_2_O_3_ or ZrO_2_ for 23 h before a 1-h incubation with a 5 mM ATP treatment. (**b**) THP-1 macrophages activated using PMA before being treated with 10 ng/mL LPS for 23 h followed by a 1-h treatment with 50 µm^3^ particles per cell Al_2_O_3_ or ZrO_2_ before cells were collected and used in conjunction with Trypan Blue in order to assess cell viability. There was a significant decrease in cell viability experienced by the cells that were treated with 50 µm^3^ particles per cell Al_2_O_3_ (P = 0.0001) or 50 µm^3^ particles per cell ZrO_2_ (P < 0.0001). (**c**) Cell supernatant was collected following treatment and ELISA performed to assess the secretion of IL-1β. In this instance, the untreated cells which received ATP only showed an increase in IL-1β secretion (P = 0.0459). LPS-treated (10 ng/mL) cells which were subject to ATP had a highly significant increase in IL-1β compared to those which received no ATP (P < 0.0001). There was a significant increase in IL-1β secretion in the Al_2_O_3_ treated group which were subject to ATP treatment (P < 0.0001). IL-1β protein secretion was significantly increased in the ZrO_2_-treatment group which received ATP compared to those which had not (P < 0.0001). (**d**) Cell supernatants were collected following treatment and used for IL-1β protein secretion analysis by ELISA. There was no significant increase in IL-1β secretion in the cells which treatment experienced a significant increase in IL-1β secretion (P = 0.0045).

Inflammasome activation was investigated by using ATP treatment to mimic a DAMP response to determine whether ceramic metal oxides could mimic PAMP-like activation of THP-1 cells as suggested through the TLR4-inhibtion experiments. Cells were treated for 23 h with 50µm^3^ particles per cell Al_2_O_3_ or ZrO_2_ before being exposed to 5 mM ATP for one hour and the supernatant collected for ELISA assessment of IL-1β protein secretion. Cells were also collected, and viability was analysed using Trypan Blue. There was significant increase in *IL-1β* gene expression (P < 0.0001) and protein secretion (P < 0.001) from cells activated with ATP post Al_2_O_3_ or ZrO_2_ treatment compared to cells that did not receive any ATP. Activated THP-1 cells were also pre-treated with LPS for 23 h and then either 5 mM ATP or 50 µm^3^ per cell Al_2_O_3_ or ZrO_2_ for 1 h before cell viability was assessed using Trypan Blue and IL-1β secretion was analysed using ELISA. There were statistically significant decreases in cell viability following treatment with LPS and ceramics (P = 0.0001 for Al_2_O_3_ and P < 0.0001 for ZrO_2_). However, no significant increase in IL-1β protein secretion was found in the cells which received Al_2_O_3_ after the LPS pre-treatment (P = 0.4912). The ZrO_2_-treated cells, on the other hand, experienced a significant increase in the secretion of IL-1β (P = 0.0045) (Fig. 6).

TLR4 blockade of THP-1 macrophages was used in order to determine the role of TLR4-mediated immunomodulation in the context of inflammasome activation. THP-1 macrophages were pre-treated with CLI-095 for 6 h and then subsequently treated with 10 ng/mL LPS as a positive control or 50 µm^3^ per cell Al_2_O_3_ or ZrO_2_, and untreated cells were used as a negative control. Protein was then extracted using RIPA buffer and total protein concentration determined before separation via gel electrophoresis and transfer of proteins to a PVDF membrane. Blots were then taken following incubation of the membrane with an anti-IL-1β antibody or anti-β-actin which acted as a control. Pro-IL-1β levels remained constant irrespective of the treatments used, however, the mature cleaved form of IL-1β was markedly increased in the LPS and ceramic groups when compared to untreated. Moreover, in the LPS and ceramic groups, those which received TLR4 blockade had substantially less of the mature IL-1β present (Fig. 6, Supplementary Material).

## Discussion

Phagocytosis of foreign material by macrophages is the first step in initiating innate immune responses in vivo. Here, we show that THP-1 macrophages are capable of phagocytosing metal ceramic oxide nanopowders in vitro which acts as the initial step of inducing an inflammatory response. Similar findings have been reported in which osteoblast cell lines are capable of phagocytosing metal debris from orthopaedic joints in vitro^31^.

Radziun et al., reported that aluminium oxide nanoparticles are capable of penetrating the membranes of the L929 mouse fibroblast cell line and the BJ human fibroblast cell line without a significant decrease in cellular viability^32^ which is analogous with the results presented here from the THP-1 human macrophage cell line. Conversely, Ye et al., reported that 3T3-E1 mouse osteoblast-like cell line when treated with ZrO_2_ nanopoarticles saw a significant decrease in cell viability as well as a significant increase in the production of reactive oxygen species^33^. Together these reports suggest that while these cell types are capable of phagocytosing Al_2_O_3_ there appears to be no significant cytotoxic effects whereas ZrO_2_ on the other hand is capable of inducing cytotoxicity. The findings presented here support findings of Radziun et al., but dispute those of Ye et al., therefore highlighting the disparity in the literature regarding ceramic metal oxides as well as the sensitivity of different cell types. It is therefore hypothesised that these ceramics may induce different effects in different cell types, hence the human macrophage THP-1 cell line did not undergo any substantial cytotoxic effects of either Al_2_O_3_ or ZrO_2_. Interestingly, there appears to be a notable difference in the immunogenicity of Al_2_O_3_ and ZrO_2_ as observed in this study which may account for the previous findings which demonstrated increased cytotoxicity of ZrO_2_. Here we demonstrate that in all the genes investigated ZrO_2_ elicited a higher level of relative gene expression than that of Al_2_O_3_ and this was reflected in the protein secretion data also presented in this study. This is something to be considered in future studies especially given that typically aluminium oxide is used in higher quantities than zirconium oxide in total joint replacement implants.

Previous work has shown that human fibroblasts increase *IL-6* gene expression following treatment in vitro with ceramics^13^. In the present study, we show that *IL-8*, *CCL2*, *CCL3*, and *CCL4* all experience significant increases in relative gene expression following ceramic treatment. As these factors are inherently pro-inflammatory and chemotactic it was also important to assess protein secretion of these factors. Similarly, to the gene expression data presented, protein secretion of IL-8, CCL2, CCL3, and CCL4 significant increased following ceramic treatments. Nakashima et al., reported that both CCL3 and CCL4 experience significant increases in gene expression and protein secretion from human primary macrophage cells following treatment with titanium-alloy wear debris^34^. This is consistent with the findings presented here following treatment with ceramic metal oxides and demonstrates that ceramic biomaterials are capable of eliciting pro-inflammatory phenotypic changes in macrophages similar to that of metal wear debris. Pro-inflammatory chemokines are key in the recruitment of cells during an immune response and in the case of CCL2, CCL3, and CCL4 increase monocyte migration. These recruited cells then mediate the inflammatory responses through further secretion of chemokines and changes to inflammatory gene expression. Mawdesley et al*.* demonstrated that in a human monocytic cell line, similar to the one utilised in this study, CCL3 and CCL4 secretion increased significant following treatment with cobalt ions^28^. Moreover, this CCL3 and CCL4 response was also attenuated using TLR4 inhibition and is consistent overall with the findings of Nakashima et al*.* and the findings presented here. Secretion of pro-inflammatory chemokines has previously been proposed as potential biomarkers by both Zhao et al., who demonstrated an elevated CCL3 concentration in patients with knee OA^25^, and Hulin-Curtis et al., who showed the importance of the presence of CCL2 in OA patient’s synovial fluid^24^. The in vitro findings presented here confirm the importance of pro-inflammatory secretions in patients with osteoarthritis, as these factors may already be elevated prior to interaction of ceramic or metal debris with innate immune cells such as macrophages which may amplify ongoing inflammatory responses in OA patients. Evidence has also shown elevated CCL3 and CCL4 protein expression using immunohistochemistry in patient samples taken at the point of revision following failure of a metal-on-metal hip replacement^28^. These findings, together with the results presented here, strongly support the notion that the induction of CCL3 and CCL4 secretion can be initiated by both ceramic and metal debris therefore leading to an increased inflammatory response. Moreover, Sterner et al. demonstrated the ability of Al_2_O_3_ to induce a fourfold increase in tumour necrosis factor alpha (TNFα) secretion when compared to an untreated control in THP-1 macrophages^35^. Although not explored in this study, TNFα is a key pro-inflammatory cytokine and can activate a number of inflammatory pathways by inducing nuclear factor kappa-light-chain-enhancer of activated B cells (NFκB) and mitogen-activated protein kinase (MAPK) activation which are both key regulators in the production of CCL2, CCL3, and CCL4. Therefore, it would be of interest to investigate TNFα secretion as well as the induction of the chemokines investigated in this study.

**Figure 7:**
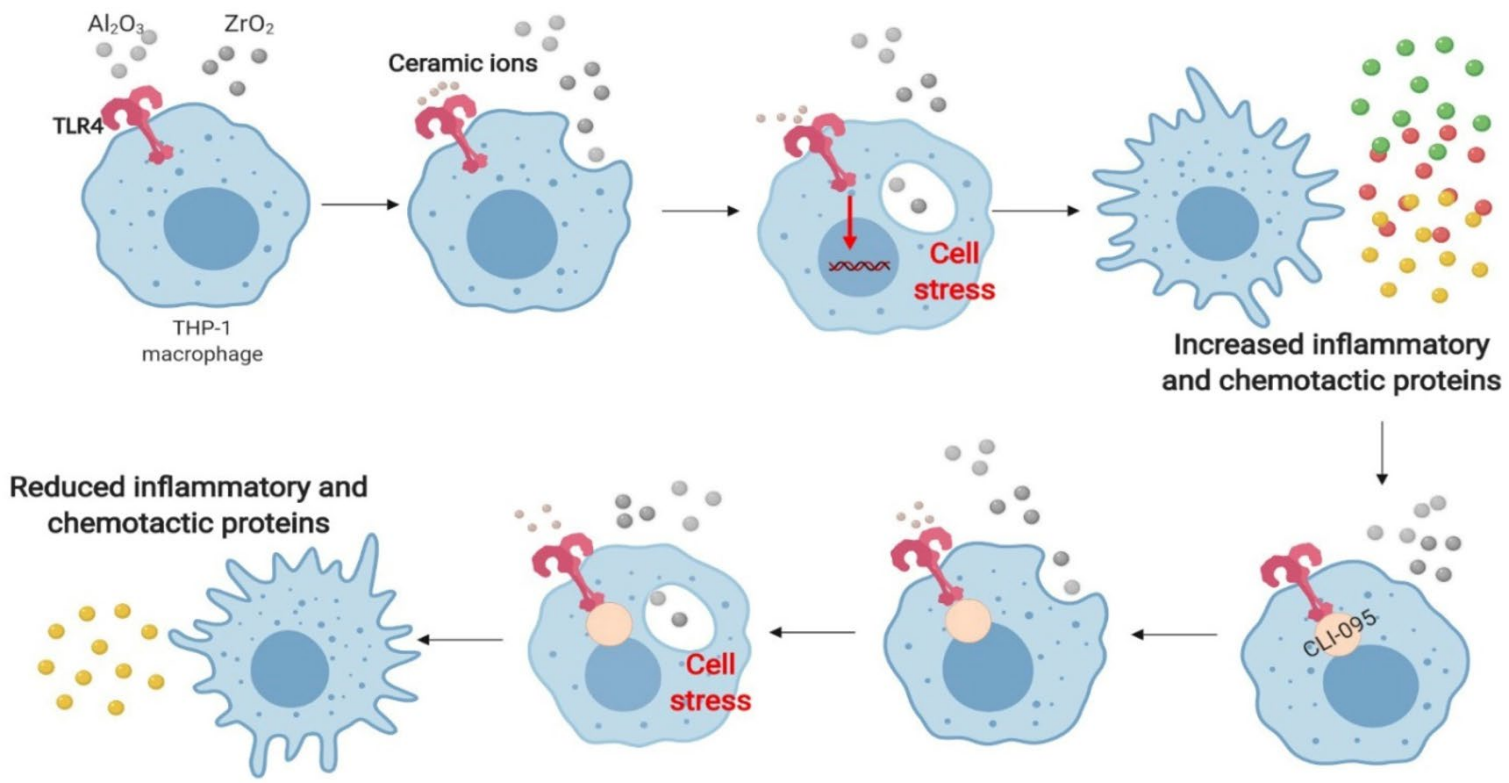
Working model of TLR4-mediated inflammation to metal oxide ceramic nanopowders. Ceramic ions released from metal ceramic oxide nanopowders activate TLR4 on macrophages. These macrophages are also capable of phagocytosing ceramic metal oxides which when combined with TLR4 activation causes cell stress and a pro-inflammatory phenotype characterised by inflammatory and chemotactic gene expression and protein secretion. CLI-095 binds intracellularly to TLR4 and prevents the recruitment of adaptor molecules for downstream signalling, therefore there is a reduced pro-inflammatory phenotype. Macrophages remain capable of phagocytosing ceramic oxide nanopowders so there is still a degree of cell stress, however, with a lack of TLR4 activation this is minimal. Image created with BioRender (https://BioRender.com).

TLR4 inhibition using CLI-095 has been investigated thoroughly and Lawrence et al. reported that TLR4 inhibition is key for blocking inflammatory responses to cobalt ions^16^. Interestingly, THP-1 macrophages which were pre-treated with CLI-095 prior to ceramic oxides showed significant decreases in *IL-8* gene expression and protein secretion compared to those which did not receive the small-molecule inhibitor. This is in agreement with the findings of Lawrence et al. and suggests a possible mechanism by which ceramic debris is capable of eliciting inflammatory responses in patients as detailed in Fig. 7. TLR4 activation by metal ions is highly specific and has been demonstrated by Tyson-Capper et al. in a study which utilised reporter cell lines which expressed either human or murine TLR4. The cell lines with murine TLR4 experienced no significant activation whereas the human TLR4-expressing cells saw significant activation at a clinically relevant dosage of cobalt ions^36^. This is in agreement with the findings reported in this study as it highlights that human TLR4 is required for cellular activation in response to metals, which are similar to the metal ceramic oxides utilised here.TLR4 has been implicated in a number of inflammatory diseases and is an attractive option for targeted therapies. Inhibition of TLR4, using statins in atherosclerosis^37^ and using a small-molecule inhibitor in septic shock^38^, demonstrates there may be translational benefit of targeting TLR4 in the future.

Inflammasome activation is a key step in the upregulation of *IL-1β* gene expression and protein secretion. However, for mature IL-1β secretion to occur there needs to be additional ‘danger’ signals which, in vivo, most likely comes in the form of damage-associate molecular patterns (DAMPs) such as ATP from lysed cells. Stoffels et al*.* reported that LPS-treated human PBMCs showed significant increases in *IL-1β* gene expression once they had been treated with ATP as a secondary stimulus^39^. This supports the findings presented here whereby THP-1 macrophages treated with LPS or ceramics for 23 h followed by an hour of ATP treatment experienced significant increases in IL-1β protein secretion. These findings, combined with the TLR4 inhibition work, suggests that ceramics can elicit pro-inflammatory immune responses through a PAMP-like mechanism in a similar manner to that of LPS and are capable of ‘priming’ the inflammasome. Desai et al*.* reported that THP-1 human macrophage cells treated with alumina for 24 h experienced a significant increase in the secretion of IL-1β which was then attenuated through the use of NADPH oxidase inhibition as well as caspase-1 inhibition. This suggests that ceramics alone, without the addition of ATP, are capable of inflammasome activation^40^. Moreover, it should be noted that in the cells which had been treated as normal with Al_2_O_3_ or ZrO_2_ followed by 5 mM ATP there was also a significant decrease in cell viability. Lima Jr et al*.* reported that lysosomal rupture is a key step in controlling the induction of NLRP3 inflammasome signalling including the induction of pyroptosis, inflammatory responses, and a mode of necrotic cell death characterised by caspase-1-independent apoptosis which could be induced by alum^41^. The lysosomal disruption investigated demonstrated a mechanism by which cell death is induced in a caspase-1-independent manner which is distinct from caspase-1-mediated pyroptosis and results in minimal secretion of IL-1β which was also observed in this study. Therefore, it could be hypothesised that in the LPS-primed cell experiments carried out in this study the mechanism by which cell death occurred what primarily necrotic and thus resulted in lower IL-1β secretion whereas the cells treated with ceramics followed by ATP were more likely to undergo pyroptosis and therefore experienced a significant increase in IL-1β secretion. However, in the LPS-primed cells it should be noted that IL-1β secretion was significantly increased in the ZrO_2_ group but not to the extent seen in the ATP treatment, therefore further investigation is required which utilises other methods such as Western blot analysis to study the role of ceramic oxides on Gasdermin D expression in order to determine whether pyroptosis or necrotic cell death is the primary mechanism by which these ceramic oxide nanopowders are capable of activating the inflammasome. TLR4 blockade using a small molecule inhibitor caused substantial decreases in the presence of IL-1β in the THP-1 macrophages treated with LPS and ceramics which supports our earlier findings which suggested that ceramic oxide nanopowders are capable of activating TLR4 in a similar manner to that of LPS. Moreover, pro-IL-1β expression levels remained constant despite treatment with LPS or ceramic oxide nanopowders which suggests that TLR4 activation is a key player in the cleavage of pro-IL-1β but not production and that other pathways may be required for full inflammasome activation including ATP, or DAMPs, required for secretion of the cleaved protein; prolonged TLR activation contributes to ATP and reactive oxygen species (ROS) levels.

While the work presented in this study supports the hypothesis of a TLR4-mediated inflammasome activation induced by ceramic biomaterials, further evidence is required in order to determine the role of the inflammasome through the targeting of specific proteins such as NLRP3. By targeting NLRP3 using commercially available inhibitors further investigation can focus on whether ceramic oxides directly activate this inflammasome mediator and whether this is what leads to the significant increase in IL-1β secretion and pyroptotic cell death experienced in vitro. Overall, these findings are particularly important as inflammatory responses which can be influenced through the upregulation of IL-1β^27^ can induce osteolysis often leading to aseptic loosening of the implant and thus the need for revision surgery so this remains a key area for further study.

Another important area to expand upon is the evaluation of the mechanism by which pro-inflammatory responses are mediated following challenge with metal ceramic oxides. As shown in Fig. 1, THP-1 macrophages are capable of the phagocytosis of these ceramics which is key for the initiation of an innate immune response. Evidence published by Yang et al*.* demonstrated the ability of metallic wear debris to induce an increase in ROS in rat osteoblast cells which lead to mitochondrial degradation and caspase-3, caspase-9, and caspase-12-dependent apoptosis^42^. It would therefore be useful to investigate the effect of ceramic metal oxides, similar to that used in joint prostheses, on the endosomal and mitochondrial degradation previously investigated. Moreover, it would be particularly interesting to consider the role of TLR4 in this process, as LPS-dependent inflammasome priming causes significant IL-1β protein secretion following activation with ATP. Future studies should aim to incorporate TLR4 inhibition into these experiments.

Another interesting factor to consider is that aluminium salts, commonly referred to as ‘alum’, are widely used as adjuvants in vaccines in order to improve outcomes relating to immunity following vaccination. As such, it has been demonstrated that NLRP3-dependent upregulation of IL-1β can be induced by alum^43^. Moreover, further investigations by Hornug et al*.,* determined that alum is able to induce this NLRP3 activation through a mechanism primarily defined by lysosomal degradation and therefore is capable of inducing the second ‘activation’ signal required for full NLRP3 activation^44^. However, it should be noted that the Al_2_O_3_ utilised in this study has a different composition and therefore further studies would be required in order to investigate the effect that aluminium salts have on THP-1 macrophage phenotype. This could be achieved in a similar manner to what has already been investigated and published by Lawrence et al*.*, with cobalt^8^ by utilising a soluble form of aluminium, in this case alum salts, in order to compare the effectiveness of particles compared to ions in stimulating THP-1 macrophages in vitro.

As this model is in vitro it is appreciated that further investigation of the immune responses to ceramics will be required before any definitive or translational conclusions can be drawn. Nevertheless, this study suggests that whilst ceramics may once have been considered as bio-inert, they are capable of eliciting pro-inflammatory responses in vitro as demonstrated in a human macrophage model. Additionally, in this study the dosages of metal ceramic oxide nanopowders are likely to be higher than would be seen in vivo but growing reports of adverse reactions to ceramic implants shows that monitoring patients over time is key in determining the volume of ceramic wear and its inflammatory impact. However, the range of concentrations of ceramic metal oxides used in this study (0.5–50 µm^3^ per cell) has been used previously and it was found that even at the highest concentration of alumina in vitro the effects were only mildly cytotoxic^45^ and therefore the same concentrations were tested in this study. Further investigations could utilise patient samples in order to have a clinically relevant dose of ceramic oxides in future experiments.

## Methods and materials

### THP-1 cell line

THP-1 cells are a human monocytic cell line derived from a one year old male with acute monocytic leukaemia (ATCC TIB-202)^46^. All cells used were negative when tested for mycoplasma contamination using the MycoAlert Mycoplasma Detection Kit (Lonza, Basel, Switzerland) according to the manufacturer’s instructions. Cells were cultured in complete RPMI-1640 medium supplemented with 10% foetal bovine serum, 50 U/mL penicillin, 50 µg/mL streptomycin, and 2 mM l-glutamine (all Sigma-Aldrich, Gillingham, UK) unilt 90–100% confluent. THP-1 cells were then seeded at a density of 500,000 into 12-well culture plates (Greiner Bio-One, Kremsmunster, Austria) and activated using 5 ng/mL phorbol 12-myristate 13-acetate (PMA) (Peprotech, London, UK) to become adherent^47^.

### Ceramic treatments

Alumina oxide (Al_2_O_3_) or Zirconium oxide (ZrO_2_) nanopowder (Sigma-Aldrich) was diluted in blank RPMI-1640 (containing no supplements) to a stock concentration of 500 µg/mL and sonicated for 10 min prior to use. Both Al_2_O_3_ and ZrO_2_ were used in conjunction with the Pierce Chromogenic Endotoxin Quant Kit (ThermoFisher Scientific, Massachusetts, USA) prior to use according to the manufacturer’s instructions in order to determine endotoxin levels present in these commercially available nanopowders. The Al_2_O_3_ and ZrO_2_ samples were both below 0.125 EU/mL of endotoxin. THP-1 cells were washed with phosphate buffered saline (PBS) and then treated with dosages of 0.5–50 µm^3^ per cell for 24 h. Untreated cells were used as a negative control and TLR4-specific LPS (from *E. coli* serotype J5, Alexis Biochemicals, San Diego, USA) diluted in complete RPMI-1640 medium to a stock concentration of 1000 ng/mL was used as a positive control at a working concentration of 10 ng/mL. LPS was used as a positive control as it is a known stimulant of inflammatory responses via the TLR4 pathway.

### Cytotoxicity assays

Adherent THP-1 cells were treated with ceramics as per the protocol above. Cells were then treated with 1 mL Accutase (Sigma-Aldrich) and incubated for 15 min in order to detach the cells from the tissue culture plate. Following incubation, the cells were collected in Eppendorf tubes and centrifuged at 5500×*g* for 5 min. The cell supernatant was removed, and the cells were resuspended in 200 μL complete RPMI-1640 medium. 10 μL of cell suspension was then mixed with 10 μL trypan blue (Sigma-Aldrich) and mixed gently. 10 μL of this mixed solution was then used on a Luna cell counting slide in conjunction with the Luna II (Logos Biosystems, Seoul, South Korea) fully automated cell counter in order to calculate percentage viability. Additional THP-1 cells were seeded at a density of 50,000 cells per well of a 96-well tissue culture plate and treated in accordance with the protocol above. The XTT Cell Proliferation Kit II (Sigma-Aldrich) was used according to the manufacturer’s instructions and absorbance read using a BioTek Synergy HT microplate reader at 450 nm.

### TLR4 inhibition

THP-1 cells were pre-treated with CLI-095 (Invivogen, San Diego, USA), a small-molecule inhibitor of TLR4, for 6 h at a concentration of 1 µg/mL before being removed. The cells were then treated with ceramics according to the ceramic treatments protocol above.

### Inflammasome priming

THP-1 cells were treated as per the ceramic treatment protocol above for 23 h before being exposed to 5 mM ATP (a known trigger of NLRP3 inflammasome activation following PAMP stimulation) (Invivogen) for 1 h to induce inflammasome activation and secretion of IL-1β. Cell viability was assessed as per the cytotoxicity protocol above.

### Pyroptosis induction

THP-1 cells were activated using PMA as per the protocol above before being primed with LPS at a concentration of 10 ng/mL for 23 h. Cells were then exposed to 50 µm^3^ per cell Al_2_O_3_ or ZrO_2_ in order to determine whether ceramic oxide nanopowders can fully activate the inflammasome. Cell viability was assessed as per the cytotoxicity protocol above and the cell supernatant was analysed for IL-1β protein secretion.

### Quantitative real-time PCR (qRT-PCR)

RNA was isolated using the ReliaPrep RNA Miniprep System (Promega, Wisconsin, USA) according to the manufacturer’s instructions. cDNA was synthesised from isolated RNA using Tetro cDNA Synthesis Kit (BioLine Meridian Bioscience, Ohio, USA) in accordance with the manufacturer’s instructions. qRT-PCR was performed using TaqMan gene expression probes (ThermoFisher) All target genes were normalised to *18S* as a housekeeping gene.

### Enzyme-linked immunosorbant assay (ELISA)

ELISA was used to quantify protein secretion following ceramic treatments. IL-8, CCL2, CCL3, CCL4, and IL-1β secretions were quantified using Human ELISA DuoSet kits (R&D systems, Minneapolis, USA) according to the manufacturer’s instructions.

### Transmission electron microscopy (TEM)

THP-1 cells were seeded and treated as per the ceramic treatments protocol above before being washed with 1 mL cold PBS and treated with 1 mL Accutase for 15 min in order to detach the cells from the tissue culture plate. Once detached, the cells were collected in Eppendorf tubes and centrifuged for 5 min at 5500×*g*. The supernatant was removed, and the cell pellet was suspended in 1 mL 2% glutaraldehyde. Samples were then processed by EM Research Services (Newcastle University) by being dehydrated through graded alcohols and then placed into propylene oxide before being embedded in epoxy resin. The samples were then cut into ultra-thin sections and stained with heavy metals in order to visualise the structural components and of the cells under TEM.

### Western blot analysis

THP-1 cells were harvested using radioimmunoprecipitation assay (RIPA) buffer and protein concentration of each sample was determined using the Bio-Rad DC protein assay kit (Bio-Rad Laboratories, Hertfordshire, UK). Equal amounts of protein were loaded on a 12% acrylamide gel and electrophoresed. The protein was then transferred to a polyvinylidene fluoride (PVDF) membrane using the Trans-Blot Turbo Transfer System (Bio-Rad Laboratories) according to the manufacturer’s instructions. The membrane was blocked for 1 h at room temperature with a 10% blocking solution (dry milk powder in TBST [tris-buffered saline + 0.1% Tween 20]). After blocking, the membrane was then incubated with anti-IL-1β (GeneTex, California, USA) or anti-β-actin (GeneTex) overnight at 4 °C before being washed with TBST and then incubated for 1 h at room temperature with the corresponding peroxidase-conjugated secondary antibody (Agilent Technologies, California, USA). Blots were washed again in TBST before being soaked in Pierce ECL Western Blotting Substrate (ThermoFisher) and exposed to x-ray film then the bands were developed.

### Statistical analysis

Statistical analysis was performed using GraphPad Prism 8.0 (GraphPad Software Inc., San Diego, USA). All error bars were calculated from the standard error of the mean (SEM). Statistical significance was analysed using one-way ANOVA with a Tukey’s post-hoc multiple comparisons test and is indicated as follows: ns = non-significant, *P < 0.05, **P < 0.01, ***P < 0.001.

## Supplementary Information

Supplementary Information.
