# Supplementary material for: Inflammatory responses to metal oxide ceramic nanopowders
Source: Sci Rep. 2021 May 18;11:10531. doi: 10.1038/s41598-021-89329-7 (PMC8131359; doi:10.1038/s41598-021-89329-7)
Supplement: Supplementary file 1 — Supplementary Information. [file 41598_2021_89329_MOESM1_ESM.docx]

**Inflammatory responses to metal oxide ceramic nanopowders**

Shannon Jamieson^1^; Amy Mawdesley^1^; David Deehan^2^, John Kirby^1^; James Holland^2^, Alison Tyson-Capper*^1^

**Supplementary Material**

**Supplementary Figure 1.** Protein expression of IL-1β following induction of inflammasome activation with our without TLR4 blockade

THP-1 cells were activated using PMA and pre-treated with CLI-095 for 6 hours before being treated with LPS (10ng/ml) or 50μm^3^ per cell Al_2_O_3_ or ZrO_2_. Protein was then extracted using RIPA buffer and separated using gel electrophoresis before being transferred to a PVDF membrane, incubated with anti-IL-1β or anti-β-actin antibodies, and then developed using electrochemiluminescence. Pro-IL-1β levels remained constant between treatments but the cleaved mature form of IL-1β was substantially increased in the LPS, Al_2_O_3_, and ZrO_2_ groups without CLI-095 compared to untreated. In all three groups, there was a marked decrease in IL-1β present in the samples which had been subjected to TLR4 blockade. All data presented here is representative of n=3.

**
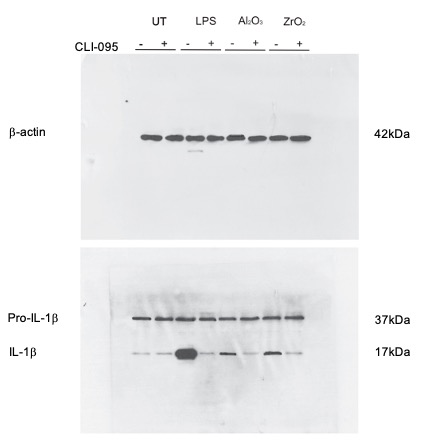
**
